# Constraints on lanthanide separation by selective biosorption

**DOI:** 10.1016/j.isci.2025.112095

**Published:** 2025-03-27

**Authors:** Carter Anderson, Sean Medin, James L. Adair, Bryce Demopoulos, Liad Elmelech, Emeka Eneli, Chloe Kuelbs, Joseph J. Lee, Timothy J. Sheppard, Deniz Sinar, Zacharia Thurston, Mingyang Xu, Kang Zhang, Buz Barstow

**Affiliations:** 1 Department of Physics, Williams College, Williamstown, MA 01267, USA; 2 Department of Biological and Environmental Engineering, Cornell University, Ithaca, NY 14853, USA

**Keywords:** Biotechnology, Chemistry

## Abstract

Lanthanides, key components of sustainable energy technologies, can be separated using microorganisms with selective biosorption capabilities that sometimes rival traditional solvent extraction methods. Recent discoveries show that single genetic mutations in *Shewanella oneidensis* can improve lanthanide biosorption selectivity, while larger genomic modifications in *Vibrio natriegens* yield greater improvements. To evaluate whether these enhancements are sufficient for industrial implementation, we developed three theoretical models of lanthanide separation by biosorption and desorption. Model 1 suggests that single-locus genetic changes could reduce separation time by 25%, while multi-locus modifications could achieve up to 90% reduction. Model 2 indicates that with multiple binding sites, larger genetic modifications would be necessary for high-purity separation. Model 3 proposes an alternative approach using multiple microbes with modest selectivity improvements: initial microbes enrich the target lanthanide, while subsequent ones remove contaminants.

## Highlights

• Three models explored lanthanide enrichment using immobilized biosorbing microbes
• Model 1 predicts that genetic edits can cut separation length from 25% to 90%.
• Model 3 predicts that high-purity separation is possible by combining multiple microbes

## Graphical abstract

**Figure:**
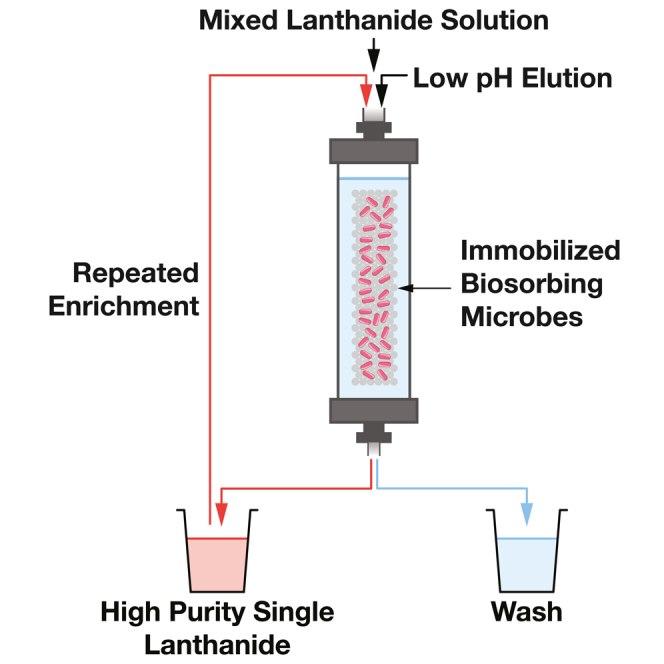

## Introduction

Rare earth elements (REEs) are essential ingredients in sustainable energy technologies like the high-strength lightweight magnets used in electric vehicles and wind turbines,^1^^,^^2^ high-efficiency lighting,^3^ nuclear reactors,^4^ rechargeable nickel-metal-hydride batteries,^5^ lightweight high-strength alloys,^6^^,^^7^^,^^8^ and high-temperature superconductors.^9^^,^^10^^,^^11^^,^^12^ Strictly, REEs refer to scandium, yttrium, and the lanthanides (lanthanum to lutetium, *Z* = 57 to 71), but often when we are talking about the challenges associated with REE, we are really talking about the challenges associated with the lanthanides. When we say REE in this article, we almost always mean it as shorthand for lanthanides.

This article is a companion piece to two recent articles from our lab on understanding the genetics of selective lanthanide biosorption^13^ and on genetic engineering of selective lanthanide biosorption.^14^ Because of the similar subjects of these three papers, their introductions are similar. In this article, we have tried to simplify the writing and include additional references, but in some cases, we simply could not find a better way to express the ideas we thought were important to convey.

Because REEs are so useful for sustainable energy technology, total demand for them could increase by 3- (under the stated policies scenario) to 7-fold (under a sustainable development scenario) by 2040.^15^ But, the extraction of REEs from ore and their subsequent purification require high temperatures and harsh solvents and leave considerable quantities of toxic waste.^16^^,^^17^^,^^18^ These processes give sustainable energy technologies reliant on REEs a high environmental impact and large carbon footprint.^19^

The chemical similarity of the lanthanides means that separations of adjacent or near-adjacent lanthanides pose an enormous challenge for conventional chemical methods, requiring multiple enrichments by organic solvent extractions in extremely long mixer settler devices.^20^^,^^21^^,^^22^^,^^23^ As a consequence of their high environmental standards, higher-income nations like the United States have no capacity to produce purified lanthanides. In fact, only two lanthanide purification plants exist outside of China.^17^^,^^18^^,^^24^

There is a growing optimism that biotechnologies could replace many of the existing methods for extracting and separating individual lanthanides. Biomining with microbes from the genus *Acidithiobacillus* already supplies 5% of the world’s gold and 15% of its copper.^25^ We anticipate that a lanthanide biomining system will operate in three steps: (1) bioleaching metals from an ore or end-of-life feedstock like a magnet, (2) separating the lanthanides from all other metals present in the leachate (e.g., uranium and thorium from an ore like monazite, or iron from a magnet), and (3) separating individual lanthanides.^13^

Significant progress has already been made in developing microorganisms for the first bioleaching step of REE biomining^26^^,^^27^^,^^28^ and in the second total lanthanide separation step.^29^^,^^30^^,^^31^^,^^32^^,^^33^^,^^34^^,^^35^^,^^36^ For example, lanmodulin, a lanthanide-binding protein discovered in methylotrophic bacteria, is selective for lanthanides in the presence of molar amounts of competing metal cations.^31^^,^^35^ Meanwhile, lanthanide-binding tags (LBTs), attached either to the surface of *Caulobacter crescentus*^30^ or an engineered curli biofilm,^32^ can selectively bind lanthanides in the presence of competing metals. Finally, both *Methylorubrum extorquens*^36^ and *E. coli* engineered with surface-displayed LBTs^33^ are able to preferentially accumulate heavy lanthanides from a mixed solution of lanthanides. Furthermore, Mattocks et al. recently demonstrated production of >98% pure solutions of neodymium and dysprosium in a single pass,^37^ while Nelson et al. have created molecules with separation factors up to 213 for neodymium/dysprosium.^38^ As a result of these breakthroughs, a lanthanide bioseparation system might not need to separate individual lanthanides in the presence of high concentrations of competing metals.

We are betting that selective biosorption and desorption from the surface of a microbial cell is the right approach to environmentally friendly individual lanthanide separations. Biosorption can provide high metal binding capacity at low cost.^39^^,^^40^ The membranes of some bacteria like *Bacillus subtilis* bind lanthanides through a single type of binding site (teichoic acids),^41^ but many other gram-negative and -positive bacteria contain multiple types of site^42^ including proteins,^43^ lipids,^44^ and polysaccharides.^45^ A summary of existing studies of biosorption of lanthanides is shown in Table 1.

**Table 1:** Summary of prior studies of lanthanide biosorption

Species	Summary	Reference
*Bacillus subtilis*	investigation of effect of disruption of cell wall synthesis genes on lanthanide biosorption	Moriwaki et al.^41^
*Galdieria sulphuraria*	selective biosorption of lanthanides from aqueous solutions	Manfredi et al.^46^
*Halomonas* sp.	demonstration of lanthanide separation with immobilized cells	Bonificio and Clarke^47^
*Magnetospirillum magneticum* AMB-1	selective biosorption and magnetic recovery of La from aqueous solutions	Mohammadi et al.^48^
*Pseudomonas aeruginosa*	selective biosorption of La, Eu, and Yb	Texier et al.^49^^,^^50^^,^^51^
*Pseudomonas* sp.	selective biosorption of La	Kazy et al.^52^
*Roseobacter* sp. AzwK-3b	demonstration of lanthanide separation with immobilized cells	Bonificio and Clarke^47^
*Shewanella oneidensis*	genetic characterization of lanthanide biosorption selectivity and demonstration of lanthanide separation with immobilized cells	Bonificio and Clarke^47^; Medin^13^
*Sphingobacterium* sp.	demonstration of lanthanide separation with immobilized cells	Bonificio and Clarke^47^
*Turbinaria conoides*	biosorption of La, Ce, Eu, and Yb	Vijayaraghavan et al.^53^
*Vibrio natriegens*	directed evolution to enhance total lanthanide biosorption and preference for heavy lanthanides	Medin et al.^14^

Bonificio et al. demonstrated lanthanide separation capability by biosorption and desorption under decreasing pH by microbes including *Roseobacter* sp. AzwK-3b and *Shewanella oneidensis* MR-1.^47^ Furthermore, Bonificio et al. demonstrated that, under specialized conditions, lanthanide separations^47^ are competitive with conventional solvent extraction. Given that no molecule is known to exist with a high separation factor for adjacent lanthanides, it is unrealistic to expect industrially acceptable purity (i.e., 95% or 99%) in a single-step biosorption and elution process. But, a separation process can (like current industrial methods) rely on repeated enrichment. We think that the low cost of biomass (relative to purified protein) could make biosorption uniquely well suited to this sort of enrichment process.

However, we want so stress that biological lanthanide separation remains an incredibly nascent technology. As a result, no single article can possibly address all of the issues that need to be tackled to industrially implement it. There is a chance (probably a good one as most technologies wind up being unsuccessful) that lanthanide separation by biosorption is not capable of providing the level of performance needed for industrial use, but we will only find this out by tackling issues as they arise in a series of articles.

Two alumni of the Barstow lab recently formed a company to commercialize REE biomining technology (REEgen, Inc.; Alexa Schmitz and Sean Medin). REEgen focuses on the first bioleaching step of REE biomining and on the third individual lanthanide separation step. A big concern of ours was that REEgen avoids trying to commercialize a technology that has fundamental scientific flaw (e.g., Theranos). While we are relatively confident in the science behind genetically engineered bioleaching,^27^^,^^28^ we were less confident about genetically engineered biosorption. Before committing to biosorption as a lanthanide separation strategy, we wanted to address its feasibility through a theoretical analysis.

We hypothesized that we could engineer lanthanide-biosorbing organisms like *S. oneidensis*^13^^,^^47^ or *Vibrio natriegens*^14^ to increase the preferential adsorption of individual lanthanides and shorten the length of a repeated enrichment process. This genetic engineering might allow biosorption to leapfrog existing lanthanide separation technologies.

In a recent work, we characterized the genetics of lanthanide biosorption by *S. oneidensis.* In total, we discovered 242 genes that control the overall level of REE biosorption and 10 that control REE-binding selectivity.^13^ The functions of these genes range from synthesis of the lipopolysaccharide layer on the outer membrane of *S. oneidensis* to assembly of mannose-sensitive hemagglutinin pilus. We hypothesized that the lanthanide-binding preference of *S. oneidensis* (or in fact any lanthanide-biosorbing microbe) can be altered by up- or downregulation of genes involved in the synthesis of structural features on its outer surfaces. However, the changes to lanthanide biosorption selectivity that we observed were small (only 1% to 4%).^13^

In another recent work, we increased the total and selective biosorption of *V. natriegens* by directed whole-genome evolution.^14^ By mutating ≈16 genomic sites (we cannot say how many precisely, but we believe about 16 out of ≈120 mutations were responsible for changing biosorption), we were able to increase total biosorption by 210%, the separation factor between lutetium and lanthanum by 49%, and the separation factor between ytterbium and thulium by 15%.

We wanted to know if mutant microbes with relatively small increases in lanthanide selectivity (either the *S. oneidensis* or *V. natriegens* mutants we now have, or future more heavily engineered variants) were employed in a lanthanide separation system, would they meaningfully improve its performance? Ultimately, this will need to be addressed through experimentation, but we felt that if a theoretical model predicted that genetic engineering would not meaningfully improve the performance of a biosorption-based separation system, then we should give up early and try something else.

In this article, we present three theoretical models of the upper-limit performance of lanthanide separation by repeated biosorption and elution and calculate the effect of small changes to lanthanide biosorption selectivity on the length of the lanthanide separation process. Furthermore, the mathematics used here can be easily applied to lanthanide separation by immobilized lanthanide-chelating proteins and peptides.

## Results and discussion

**Figure 1:**
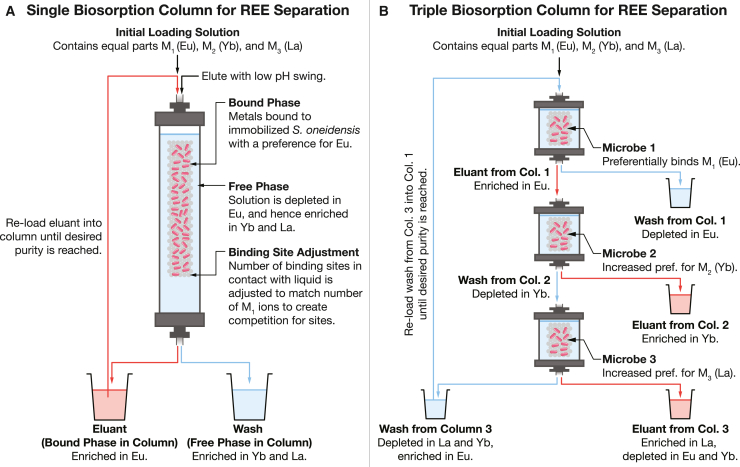
Proposed schemes for lanthanide separation by biosorption and elution Parameters for both models are shown in Table 2. (A) Single-column scheme. The performance of this scheme for an immobilized microbe with a single type of binding site (model 1; see flow chart in Figure 2) is shown in Figure 3 and summarized in Table 3; and the performance with a microbe with three types of binding site (model 2; see flow chart in Figure S1) is shown in Figure 4 and summarized in Table 4. (B) Triple column scheme (model 3; see flow chart in Figure S2). We propose that this scheme should be used when the purity of the target metal (M_1_) plateaus before reaching our desired target. Performance of the three-column scheme is shown in Figure 5 and summarized in Table 5.

**Figure 2:**
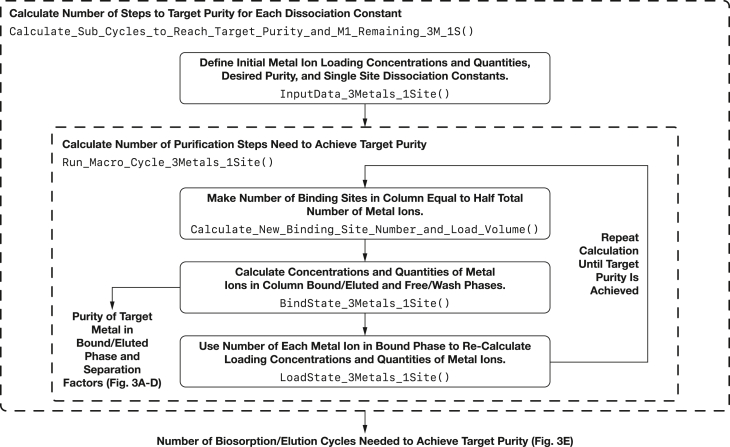
Calculation flow chart for the single-site, single-microbe model (model 1) This flow chart highlights important calculation steps in the single-site, single-microbe model (results shown in Figure 3) and the functions used to implement them in the ConcentrationSolverUtils9.py module included in the ree-purification repository.^54^ Corresponding flow charts for the three-site, single microbe model (model 2); and the three-site, three-microbe model (model 3) is shown in Figures S1 and S2.

We envision a separation process where a mixed solution of lanthanides is repeatedly biosorbed and eluted from immobilized biosorption microbe (such as *S. oneidensis* or *V. natriegens*) (Figure 1). The separation system consists of one or more chromatographic columns containing immobilized biomass (e.g., microbes on a filter,^47^ on a biofilm on a solid support, or encapsulated in gel beads^34^). Bonificio and Clark demonstrated that two cycles of a process like this could enrich for heavy lanthanides^47^ and was competitive with conventional solvent extraction methods. Flow charts illustrating the calculation processes for this theory are shown in Figures 2 (for model 1), S1 (for model 2), and S2 (for model 3). A full list of all mathematical symbols used in this article is shown in Table S1.

To sanity check the connection between experimental data and our models, we calculated the lanthanide binding per cell and per gram of dry weight of *S. oneidensis* and compared with literature values (Note S1 and Table S2). We calculate the approximate loading capacity of *S. oneidensis* to be 16.1 fg cell^−1^ or 470 fg dry weight cell^−1^ ≈ 30 mg g^−1^ of dry weight. This number compares very well to literature values of metal binding per unit mass of dry biomass assembled in Table S2.

In our model, the system is loaded with a solution initially containing equimolar quantities of three metals (M_1_, M_2_, and M_3_; e.g., Eu, Yb, and La) in a volume *V*_load_. The column contains *n*_B,T_ binding sites. After equilibration, the free fraction (the liquid) is removed from the column and moved to a wash collection container. Next, the bound fraction is eluted (for example, by a pH swing^47^^,^^55^). The eluant is then pH adjusted (to compensate for the pH swing) and its volume adjusted (to compensate for any differences in the loading and elution volume), where it can be loaded into the same or a different column. At each column step, the number of binding sites is adjusted (reduced) to be equal to half of the total number of metal ions. Note that this decision is slightly different from the one we adopted in the study by Medin et al.^13^ where the number of binding sites is adjusted so that half of all REEs are biosorbed. Although these two options produce extremely similar results under the solution conditions and dissociation constants used in this article, they will diverge in when the REE concentration drops significantly below the dissociation constant. In an upcoming work, we will explore different options for this decision. This process of load, bind, elute, and re-load is repeated until the desired purity of M_1_ in the eluant is achieved (*f*_M1,b_ = 0.9, 0.95, or 0.99). While the effect of the selective biosorption on the purity of a target metal (say M_1_) is small in any individual step, the effect of successive enrichment is not. Numerical models of this process are implemented in the Python code ree-purification.^54^

The prospects of achieving high lanthanide purity by repeated biosorption and elution depend on the nature of the binding sites on the biosorption microbe’s surface. As of the time of writing, we cannot uniquely specify all of the molecular details of lanthanide binding. As a result, we cannot perfectly predict the behavior of a repeated biosorption and elution process using the microbe. However, we can fit the preferences seen in our earlier work^13^ to two useful models (a single-site [model 1] and a triple-site [model 2] model), and then calculate the future behavior as the solution is enriched.

For the sake of simplicity, we have considered the separation of three representative lanthanides (also, at the time of writing, we had the most reliable data for them): lanthanum (La, the lightest lanthanide), ytterbium (the second heaviest lanthanide, and the heaviest lanthanide for which we have binding data), and europium (Eu, a middle lanthanide). Furthermore, we have chosen Eu as the target metal for purification as wild-type *S. oneidensis* has a preference for it under low ionic strength, high lanthanide concentration.^13^

**Figure 3:**
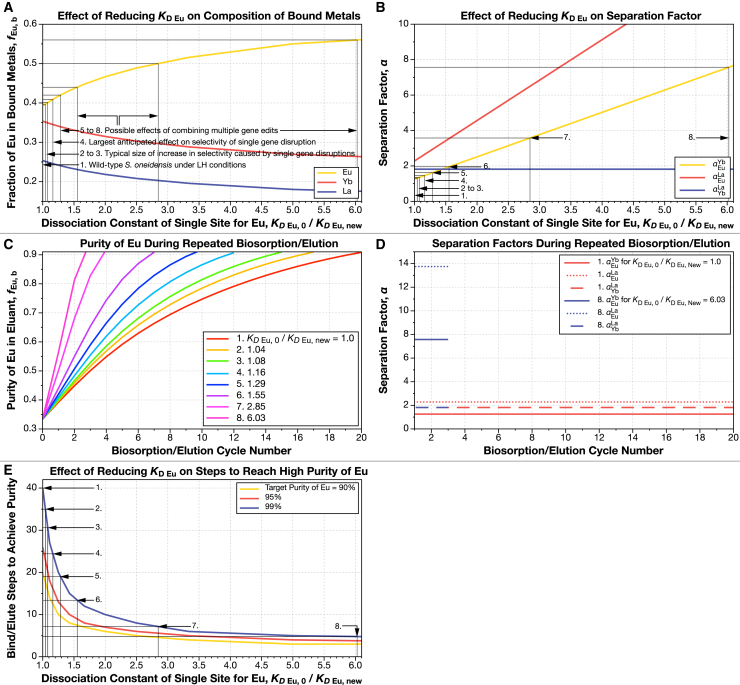
Lowering the dissociation constant of the single-site, single-microbe model (model 1) for M_1_ (Eu) dramatically reduces the number of biosorption/elution cycles needed to achieve high purity We fitted data on the fractions of each lanthanide (La, Eu, and Yb) bound by the wild-type *S. oneidensis* under low ionic strength, high REE concentration conditions to a single-binding-site model with the Fit_KD.py program in the ree-purification repository.^54^ We model the effect of reducing the dissociation constant of this single site for just Eu, while keeping the dissociation constants for Yb and La the same. (A) Reducing the Eu dissociation constant of the REE-binding site raises the fraction of Eu initially bound to the surface of *S. oneidensis*. Note that the *x* axis shows the inverse of dissociation constant for Eu. We have marked several notable binding fractions on the plot (scenarios 1 to 8 in Table 3). Wild-type behavior (scenario 1) is at the far left of the plot. For example, an increase in the fraction of Eu bound to *S. oneidensis* by 1% from 39.3% to 39.7% corresponds to a reduction in the dissociation constant by a factor of 1.04 (scenario 2). An increase in *f*_Eu, b_ to 40.9% (a 4% increase over wild type) corresponds to a reduction in *K*_D Eu_ by a factor of 1.16 (scenario 4). (B) Reducing the dissociation constant for Eu raises the Eu-Yb and Eu-La separation factors but does not affect the Yb-La separation factor. (C) Reducing the dissociation constant for Eu decreases the number of biosorption/elution cycles needed to reach high purity. Each curve corresponds to one of the example dissociation constants highlighted in (A) and (B) and Table 3. (D) The separation factors for Eu and Yb, Eu and La, and Yb and La all remain constant throughout the separation process. We have highlighted the highest and lowest example dissociation constants (scenarios 1 and 8) from (A). Note that for clarity, the colors in (D) do not correspond to the colors in (C). (E) Even small reductions in the dissociation constant of the binding site for Eu make large reductions in the number of biosorption/desorption cycles needed to achieve high purity. For example, if the dissociation constant is lowered by a factor of just 1.04 (scenario 2), the number of cycles needed to reach 99% purity drops from 40 cycles to 36. If the dissociation constant is lowed by a factor of 1.08 (scenario 3), then the number of cycles drops to 31. Panels in this figure can be reproduced with the programs Fig-3A-B.py, Fig-3C-D.py, and Fig-3E.py in the ree-purification repository.^54^ See Figures 1A and 2, Table 2, and STAR Methods section for a full description of this model.

In model 1 (Figure 1A), the microbe uses a single type of binding site that is described by three dissociation constants (e.g., for Eu, Yb, and La). A mathematical description of this model is included in STAR Methods, and a flow chart for the calculation is shown in Figure 2. The preferences of the microbe for individual lanthanides are tuned by changing the relative values of the dissociation constants (Figures 3A and 3B).

**Figure 4:**
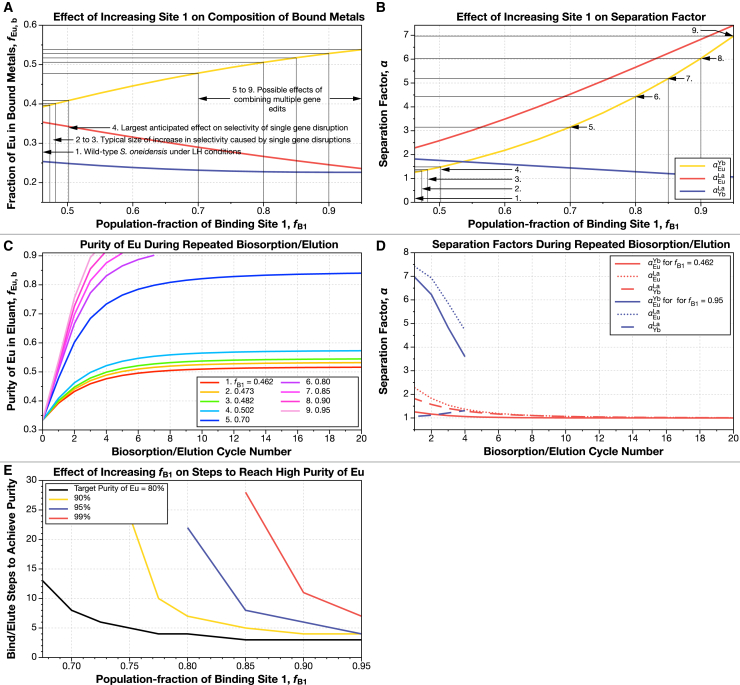
Raising the population fraction of the Eu-preferring site (site 1) in the three-site, single-microbe model (model 2) on the microbe’s surface increases the initial fraction of Eu bound but does not allow high purity to be achieved until the population fraction is raised above 90% We fitted data on the fractions of each lanthanide (La, Eu, and Yb) bound by the wild-type *S. oneidensis* under low ionic strength, high REE concentration conditions to a three-binding-site model with the Fit_fB_3S.py program. The model contains three binding sites, each with a fixed preference for one of the three REE in solution. The fit to the observed binding fractions of *S. oneidensis* is achieved by adjusting the population ratios of these sites. (A) Raising the population of the Eu-preferring site (site 1) increases the initial fraction of Eu bound to the microbe’s surface. We have marked several notable binding fractions on the plot (see Table 5). For example, an increase in the fraction of Eu bound to the microbe by 1% from 39.3% to 39.7% (scenario 2) corresponds to an increase in the population of the Eu site from 46.2% to 47.3%. An increase in *f*_Eu, b_ to 40.9% (a 4% increase over wild type; scenario 4) corresponds to an increase in the population of the Eu site to 50.2%. (B) All separation factors change as the population fractions of the three metal-binding sites change. (C) Increasing the population fraction of the Eu-preferring site decreases the number of biosorption/desorption cycles needed to increase Eu purity. However, unlike in the single-site model (Figure 3), most of the curves plateau well before reaching high purity. Each curve corresponds to one of the example dissociation constants highlighted in (A) and (B) and summarized in Table 4. (D) Unlike in the single-site model, the Eu-Yb, Eu-La, and Yb-La separation factors do not remain constant throughout the separation process. We have highlighted the highest and lowest example dissociation constants (*f*_b Eu, 39.3%_, and *f*_b Eu, 53%_) from (A). (E) High purity of Eu is possible, but increasing the population fraction of the Eu-preferring site above 90% is necessary to do it. Panels in this figure can be reproduced with the programs Fig-4A-B.py, Fig-4C-D.py, and Fig-4E.py in the ree-purification repository.^54^ See Figures 1A and S1, STAR Methods, and Table 2 for full description of this model.

In model 2 (also Figure 1A), the microbe uses three binding sites. The model contains a Eu-preferring site, a Yb site, and a La site. A mathematical description of this model is included in STAR Methods, and a flow chart for the calculation is shown in Figure S1. In this model, we keep the dissociation constants of each site (one for each lanthanide) constant, and we tune the overall lanthanide preferences of the microbe by changing the population fractions of the three sites (Figures 4A and 4B).

In model 3 (Figure 1B), we consider three microbes, each of which uses a different composition of three different binding sites to give it a preference for one of the three lanthanides considered in this model. A flow chart for the calculation is shown in Figure S2.

We can then ask what happens in a separation process if we change the parameters of the models to predict the effect of the small selectivity changes conferred by single gene modifications. Finally, we can ask what happens if we further change the parameters of the models to predict the effects of more extensive genomic editing like that seen in our later work on *V. natriegens*.

### Model 1: Fitting single-site-type binding model to experimental data

We used a minimization algorithm included in the SciPy package^56^ to fit two dissociation constants to the binding data for the wild-type *S. oneidensis*^13^ (note, we estimate that *K*_D, Eu_ ≈ 10^−6^ M). Also, we formulated this theory when we had just had data for *S. oneidensis*, so this article largely focuses on it rather than *V. natriegens*. This program can be found in Fit_Kd.py script in the ree-purification repository.^54^ We denote Eu, the metal with the highest concentration in the bound phase, as M_1_; Yb, the second highest concentration in bound phase, as M_2_; and La, the lowest concentration, as M_3_. We find, that relative to the dissociation constant for Eu (M_1_),

(Equation 1) $$K_{D,\text{Yb}}=1.256\times K_{D,\text{Eu}}$$

(Equation 2) $$K_{D,\text{La}}=2.282\times K_{D,\text{Eu}}$$

Thus, for a single-site system where the separation factors depend only upon the relative values of dissociation constants,

(Equation 3) $$\begin{array}{ll} \alpha_{\text{Eu}}^{\text{Yb}} & =\frac{K_{D,\text{Yb}}}{K_{D,\text{Eu}}}\\ & =1.256\text{,} \end{array}$$

(Equation 4) $$\begin{array}{ll} \alpha_{\text{Eu}}^{\text{La}} & =\frac{K_{D,\text{La}}}{K_{D,\text{Eu}}}\text{,}\\ & =2.282\text{,} \end{array}$$

(Equation 5) $$\begin{array}{ll} \alpha_{\text{Yb}}^{\text{La}} & =\frac{K_{D,\text{La}}}{K_{D,\text{Yb}}}\text{,}\\ & =1.817\text{.} \end{array}$$

With estimates for the apparent relative dissociation constants of Eu, Yb, and La, we can model the effects of improving the specificity of *S. oneidensis* for one of the metals. In Figure 3A, we reduce the dissociation constant of the binding site for Eu to 1/6^th^ of its original value, while holding its values constant for Yb and La. Reducing the dissociation constant of the site for Eu increases its binding fraction. In Figure 3A, we have marked several example binding fractions of Eu and their corresponding apparent dissociation constant for Eu (summarized in Table 2). For example, if the binding fraction of Eu rises from its original value of 39.3% by 1% (the size of some of the smaller selectivity changes seen in the study by Medin et al.^13^) to 39.7%, this corresponds to a decrease in the dissociation constant of the binding for Eu to 0.96 × its original value (or *K*_D Eu, 0_/*K*_*D*__Eu, New_ = 1.010). An increase in the binding fraction of Eu to 40.9% (a 4.1% increase, corresponding to one of the larger increases seen in our upcoming work by Medin et al.^13^) decreases the dissociation constant for Eu to 0.86 × its original value (or *K*_D Eu, 0_/*K*_*D*__Eu, New_ = 1.163). If we could increase the binding fraction of Eu to 56%, this would correspond to a decrease in the dissociation constant for Eu to 0.17 × its original value (or *K*_D Eu, 0_/*K*_*D*__Eu, New_ = 6.027). The corresponding separation factors for Eu and Yb; Eu and La; and Yb and La are shown in Figure 3B. As should be expected, the separation factors for Eu and Yb, and Eu and La increase as the dissociation constant for Eu is reduced, while the separation factor for Yb and La remains constant.

**Table 2:** Model parameters used in this article

Parameter	Symbol	Unit	Value
**Common parameters**			

Initial number of binding sites	*n*_B,T_	Mol	3.5 × 10^−8^
Initial number of M_1_ (Eu) ions	*n*_M1,T_	Mol	2.4 × 10^−8^
Initial number of M_2_ (Eu) ions	*n*_M2,T_	Mol	2.4 × 10^−8^
Initial number of M_3_ (Eu) ions	*n*_M3,T_	Mol	2.4 × 10^−8^
Loading volume	*V*_load_	L	4 × 10^−4^

**Single-site model (model 1)**			

Dissociation constant of single site for Eu	*K*_D, Eu_	Mol L^−1^	10^–6^
Dissociation constant of single site for Yb	*K*_D, Yb_	Mol L^−1^	1.256 × 10^−6^
Dissociation constant of single site for La	*K*_D, La_	Mol L^−1^	2.282 × 10^−6^
Number of binding sites	reduces to half total number of metal ions at each step		

**Three-site model (model 2)**			

Initial fraction of binding site 1	*f*_B1_	#	0.4618
Initial fraction of binding site 2	*f*_B2_	#	0.3703
Initial fraction of binding site 3	*f*_B3_	#	0.1679
Dissociation constant of site 1 for Eu	*K*_D1, Eu_	Mol L^−1^	10^−6^/8
Dissociation constant of site 1 for Yb	*K*_D1, Yb_	Mol L^−1^	10^–6^
Dissociation constant of site 1 for La	*K*_D1, La_	Mol L^−1^	10^–6^
Dissociation constant of site 2 for Eu	*K*_D2, Eu_	Mol L^−1^	10^–6^
Dissociation constant of site 2 for Yb	*K*_D2, Yb_	Mol L^−1^	10^−6^/8
Dissociation constant of site 2 for La	*K*_D2, La_	Mol L^−1^	10^–6^
Dissociation constant of site 3 for Eu	*K*_D3, Eu_	Mol L^−1^	10^–6^
Dissociation constant of site 3 for Yb	*K*_D3, Yb_	Mol L^−1^	10^–6^
Dissociation constant of site 3 for La	*K*_D3, La_	Mol L^−1^	10^−6^/8
Number of binding sites	reduces to half total number of metal ions at each step		

**Three-site, three-microbe model (model 3)**			

Number of binding sites on microbe 1	reduces to total number of Eu ions at each step		
Number of binding sites on microbe 2	reduces to total number of Yb ions at each step		
Number of binding sites on microbe 3	reduces to total number of La ions at each step		

See also Table S1.

We believe that model 1 is probably only applicable to microbes like *Bacillus subtilis* that use a single type of binding site (teichoic acids) for lanthanide binding,^41^ or for microbes like *Caulobacter crescentus* that have been engineered to surface-display a single type of LBTs.^30^ However, given that very little theoretical attention has been paid to separation by biosorption, we believe that it is important to start with a very simple model that is applicable to some systems. Chang et al.^57^ modeled selective biosorption of lanthanides using immobilized LBTs, but again only modeled a single pass through a column, making comparison with our theory difficult. Likewise, while Bonificio et al.^47^ did perform an enrichment of lanthanides with immobilized biosorbing cells (*Roseobacter* sp. AzwK-3b, *S. oneidensis*, *Sphingobacterium* sp., and *Halomonas* sp.), this was a much more complex solution (containing all 14 lanthanides), and only performed two enrichment steps, also making comparison with our work difficult.

### Model 1: Effect of improvement of single-site binding specificity on REE separations

We used the dissociation constants for Eu, Yb, and La (Equations 1 and 2) to calculate the separation behavior of the single-site model. This model is always able to achieve arbitrarily high purity (Figures 3C–3E). If the single-site model representing wild-type *S. oneidensis* repeatedly binds and elutes an initially equimolar solution of Eu, Yb, and La, it would take 20 cycles to reach 90% purity of Eu, 26 cycles for 95% purity, and 40 cycles for 99% purity (Figure 3E; Table 3).

**Table 3:** Summary of changes to REE biosorption selectivity on REE binding and separation with a single-site-type binding model (model 1)

1. Scenario	2. Single-site *K*_D Eu, 0_/*K*_*D*__Eu, New_	3. Initial purity of Eu	4. Increase in purity of Eu in bound phase over wild type	5. *α*_Eu_^Yb^	6. Single-site, 90% purity	7. Single-site, 95% purity	8. Single-site, 99% purity
1	1.000	0.393	1.000	1.256	20	26	40
2	1.043	0.397	1.010	1.310	18	23	36
3	1.081	0.401	1.020	1.358	16	21	31
4	1.163	0.409	1.041	1.461	13	17	25
5	1.286	0.420	1.069	1.615	10	13	19
6	1.552	0.440	1.120	1.950	8	9	14
7	2.847	0.500	1.272	3.576	5	6	8
8	6.027	0.560	1.425	7.572	3	4	5

This table summarizes the results of Figure 3. (1) Scenario entries correspond to marked points on Figure 3. (2) Fold decrease in the dissociation constant of the binding site for Eu. (3) The fraction of Eu in the metals bound to the cell surface from an equimolar solution of REE (i.e., the microbes are presented with a solution containing 33.3% Eu, 33.3% Yb, and 33.3% La, but the metals bound to the wild type (1^st^ row) consists of 39.3% Eu. (4) Fractional increase of the amount of Eu in the bound phase (*f*_Eu, b_) relative to wild type. (5) The separation factor between Eu and Ysb. (6–8) Number of biosorption/elution cycles needed to reach 90%, 95%, and 99% purity of Eu. This table can be reproduced with the programs Fig-3A-B.py, Fig-3C-D.py, and Fig-3E.py in the ree-purification repository.^54^

In the single-site model, even mutants that make small changes to the initial binding fractions of metals make big changes to separation process length (Figures 3C–3E; Table 3). If we increase the initial binding fraction of Eu by 1% (from 39.3% to 39.7%; a fairly typical increase produced for example by disruption of the *wzz* gene^13^), this corresponds to a 1.04-fold reduction in the Eu dissociation constant (scenario 2 in Figure 3A). If this mutant were used in repeated enrichment, then only 36 biosorb/elute cycles would be needed for 99% purity (Figures 3C and 3E; Table 2). A 2% increase in initial binding fraction (e.g., comparable to disruption of the *wbpA* gene^13^) drops the steps to 99% Eu purity to 31. If we could achieve a 4% increase in initial preference for Eu (the largest size of change we see due to single-gene disruptions), then we could reduce the process length to 25 steps. Increasing the initial binding preference to 50% Eu (e.g., by combining mutations to multiple genomic loci in a single strain), we could reduce the steps to 99% purity to only 8 (Figures 3C and 3E; Table 2).

### Model 2: Fitting three-site-type binding model to experimental data

In model 2 (Figures 1A and S1, STAR Methods), we modeled the selectivity of *S. oneidensis* by changing the population fraction of three binding sites that have a fixed dissociation constant for each of the metals considered in this theory (i.e., a site for Eu, La, and Yb). To perform this fit, we used a minimization algorithm using the SciPy package to fit data for the wild-type *S. oneidensis* from our recent work.^13^ This algorithm can be found in the Fit_fB_3S.py in the ree-purification repository.^54^

The first site has a preference for Eu (note that the choice of 8 was somewhat arbitrary, but it does allow us to have similarly sized population fractions for sites 1, 2, and 3) and is consistent with our single-site model (model 1; i.e., as they should be, the two models are indistinguishable in the first biosorption step),

(Equation 6) $$\begin{array}{ll} K_{D1,\text{Eu}}= & \frac{K_{D0}}{8.0}\text{,}\\ K_{D1,\text{Yb}}= & K_{D0}\text{,}\\ K_{D1,\text{La}}= & K_{D0}\text{.} \end{array}$$

The number of site 1 molecules, as a fraction of the total,

(Equation 7) $$f_{B1}=0.4618\text{.}$$

The second site has a preference for Yb,

(Equation 8) $$\begin{array}{ll} K_{D2,\text{Eu}}= & K_{D0}\text{,}\\ K_{D2,\text{Yb}}= & \frac{K_{D0}}{8.0}\text{,}\\ K_{D2,\text{La}}= & K_{D0}\text{.} \end{array}$$

The number of site 2 molecules, as a fraction of the total,

(Equation 9) $$f_{B2}=0.3703\text{.}$$

Finally, the third site has a preference for La,

(Equation 10) $$\begin{array}{ll} K_{D3,\text{Eu}}= & K_{D0}\text{,}\\ K_{D3,\text{Yb}}= & K_{D0}\text{,}\\ K_{D3,\text{La}}= & \frac{K_{D0}}{8.0}\text{.} \end{array}$$

The number of site 3 molecules, as a fraction of the total

(Equation 11) $$f_{B3}=0.1679\text{.}$$

Although the separation factors in the first separation step of the three-site model do not result from simple ratios of dissociation constants (STAR Methods), the overall numerical results are the same as in Equations 3, 4, and 5.

Intuitively, we would expect that as we increase the fraction of binding site 1 (the site with a preference for Eu), the fraction of Eu bound to *S. oneidensis* should increase. In Figure 4A, we increase the fraction of site 1 from its original value (46.3%) to 95%, while keeping all dissociation constants the same. As the fraction of site 1 increases, the fractions of sites 2 and 3 are reduced but maintain their original size ratio. For example, in the original scenario that mimics the wild-type behavior, sites 2 and 3 occupy 53.7% of the total sites (100%–46.3%). Thus, site 2 occupies 69% of the remaining sites, and site 3 occupies 31% of the remainder. As the fraction of site 1 increases, the fraction of sites 2 and 3 in the remainder remains constant. So, when *f*_B1_ = 95%, *f*_B2_ = 3.4% (69% of 5%), while *f*_B3_ = 1.6% (31% of 5%).

In Figure 4A, we have marked several example binding fractions of Eu (see Table 4 for a summary) and the corresponding calculated population fraction of site 1 (the site with a preference for Eu). For example, if the binding fraction of Eu (*f*_Eu, b_) rises from its original value 39.3% by 1% (the size of some of the smaller selectivity changes seen in the study by Medin et al.^13^) to 39.7%, this corresponds to an increase in the population of binding site 1 to 47.3%.

**Table 4:** Summary changes to REE biosorption selectivity on REE separation with a three-site-type binding model (model 2)

1. Scenario	2. Population fraction of Eu site, *f*_b, Eu_	3. Initial purity of Eu	4. Increase in purity of Eu in bound phase over wild type	5. *α*_Eu_^Yb^	6. Single-site, 80% purity	7. Single-site, 90% purity	8. Single-site, 95% purity	9. Single-site, 99% purity
1	0.463	0.393	1.000	1.263				
2	0.473	0.397	1.010	1.314				
3	0.482	0.401	1.020	1.369				
4	0.502	0.409	1.041	1.484				
5	0.700	0.478	1.217	3.152	8			
6	0.800	0.506	1.287	4.424	4	7	22	
7	0.850	0.517	1.317	5.183	3	5	8	28
8	0.900	0.528	1.344	6.030	3	4	6	11
9	0.950	0.538	1.369	6.968	3	4	4	7

This table summarizes the results of Figure 4. (1) Entries correspond to marked points on Figure 4. (2) Population fraction of the Eu-preferring site on the surface of *S. oneidensis*. (3) Calculated fraction of Eu in the metals bound to the cell surface from an equimolar solution of REE (i.e., the microbes are presented with a solution containing 33.3% Eu, 33.3% Yb, and 33.3% La, but the metals bound to the wild-type [1^st^ row] consist of 39.3% Eu). (4) Calculated fractional increase of the amount of Eu in the bound phase. (5) Calculated separation factor between Eu and Yb. (6–9) Number of biosorption/elution cycles needed to reach 80%, 90%, 95%, and 99% purity of Eu. If no number is listed, then purity cannot be achieved. This table can be reproduced with the programs Fig-4A-B.py, Fig-4C-D.py, and Fig-4E.py in the ree-purification repository.^54^

An increase in the binding fraction of Eu to 40.9% (a 4% increase, corresponding to one of the larger increases in the study by Medin et al.^13^) corresponds to an increase in the population of binding site 1 to 48.2%. The corresponding separation factors for Eu and Yb; Eu and La; and Yb and La are shown in Figure 4B. Unlike in Figure 4B, the separation factor for Yb and La does not remain constant.

### Model 2: Effect of improvement in three-site model on REE separations

What does the increase in the fraction of binding site 1 (the site with a preference for Eu) mean for the purification of Eu? If we repeatedly pass the eluant of the biosorption column through it, we should expect to enrich the solution for Eu. A simulation of this process is shown in Figure 4C. As the eluant solution is passed through the column, the purity of Eu does increase. However, unlike in Figure 3C (where the purity of Eu does always eventually reach the target of 90%), for lower fractions of site 1, the purity of Eu almost plateaus well before reaching the target purity. This is because in the three-site model, unless you can completely eliminate them, there are always places on the surface of the microbe for Yb and La to strongly bind. Unlike in model 1, where any change to the microbe is useful and reduces the steps to get to 99% purity, when there are multiple sites that are strongly selective for a single lanthanide, you need to eliminate a very high fraction of them to make a useful microbe. For example, even if the fraction of site 1 is raised from 46.3% to 80%, we will require 22 bind/elute cycles to reach 95% purity (Figure 4E; Table 4). Thus, if a biosorption microbe behaves like model 2 (i.e., it has multiple binding sites), then it is likely that extensive genetic editing will be needed to achieve high purity of Eu.

### Model 3: Three microbe separation system

The key result of our work on the genetics of lanthanide biosorption by *S. oneidensis* is that there are few, if any, dominant players in this process.^13^ Instead of one dominant type of binding site like teichoic acids in *Bacillus subtilis*, many different types of sites, encoding structural features ranging from pilus to lipopolysaccharides to outer membrane proteins, contribute to the adsorption of REE to the *S. oneidensis* membrane. This result suggests that in a successive enrichment process, *S. oneidensis* has the potential to behave like the three-site model (model 3; STAR Methods, Figures 4 and S1) and might produce plateauing purity before an industrially acceptable level is reached.

However, even if *S. oneidensis* does produce plateauing purity in a repeated enrichment process, our model of REE separation indicates that this problem is not insurmountable. The model suggests that a process that uses multiple microbes in series (Figures 1B and S2; one to enrich for the target lanthanide, and others to remove the competing lanthanides) could achieve industrially acceptable purity with only small genetic edits.

**Figure 5:**
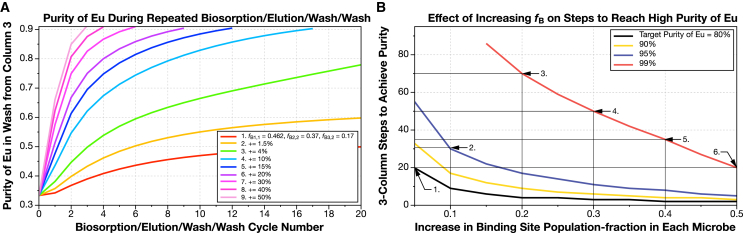
High purity of Eu can be achieved without large changes to the population faction of the Eu-preferring site if a three-column, three-microbe system (model 3) is used As we noted in Figures 4C and 4E, in contrast to using a microbe with a single type of binding site, repeated biosorption and desorption using a microbe with three binding sites does not always produce increasing purity of Eu, unless the population fraction of the Eu site is very high. To solve this problem, we turned to system where three columns each containing a different type of microbe is used in series (see Figure 1B). The first separates Eu, while the second and third steps remove Yb and La, respectively. Using this system with the wild-type microbes increases the purity of Eu slowly, but even modest changes to the population fractions of one binding site in each of the microbes produce very notable reductions in steps to high purity. Results are summarized in Table 5. (A) The purity of Eu increases faster as we increase the population fraction of the target site on each microbe. For example, + = 1.5% means that we increment the population of the Eu site on the first microbe by 1.5% (e.g., from 0.462 to 0.477), increase the population of the Yb site on the second microbe by 1.5% (e.g., from 0.370 to 0.385), and increase the population fraction of the La site on the third microbe by 1.5% (e.g., from 0.168 to 0.183). (B) Increasing the population fraction of each of the three sites in each of the three microbes produces rapid improvements in the number of steps to high purity. Panels in this figure can be reproduced with the programs Fig-5A.py and Fig-5B.py in the ree-purification repository.^54^ See Figures 1B and S2, STAR Methods, Table 2, and the theory section for full description of this model.

When used in three-microbe scheme, the three-site model representing wild-type *S. oneidensis* is only able to achieve ≈50% Eu purity (Figure 5A). However, if the population fractions of the target binding sites on each microbe are incremented by just 1.5%, then performance improves dramatically (i.e., the population of the Eu-preferring site on microbe 1 is increased from 46.2% to 47.7%, the fraction of the Yb site on microbe 2 from 37.0% to 38.5%, and the La site in microbe 3 from 16.8% to 18.3%). If we increment the population fraction of the target sites by 4% in each microbe (scenario 3 in Figure 5A), the purity of Eu starts to take-off. Notably, these increases in population fraction are typical of single genomic loci mutations (see Table 3 for connection).

The three-microbe system allows us to reach high purity of Eu by repeated biosorption and elution without heroic genetic engineering efforts. In order to reach 99% purity, we estimate that population fraction of the important site in each microbe must be incremented by ≈15%. The number of steps (in this case passes through the three-microbe system) to reach 90%, 95%, and 99% purity are shown in Figure 5 and Table 5. This suggests that this three-microbe column system could be made to operate by combining together microbes with edits to only one or two genetic loci.

**Table 5:** Summary changes to REE biosorption selectivity on REE separation with a three-microbe, three-site-type binding model (model 3)

1. Scenario	2. Increase in population of primary binding site	3. 80% purity Eu	4. 90% purity Eu	5. 95% purity Eu	6. 99% purity Eu
1	0.05	20	33	55	
2	0.10	9	17	30	
3	0.20	4	9	17	70
4	0.30	3	6	11	50
5	0.40	2	4	8	35
6	0.50	2	4	6	21

This table summarizes the results of Figure 5. (1) Entries correspond to marked points on Figure 5. (2) Increase in the population fraction of the targeted metal-preferring site on each microbe. For example, + = 5% means that we increment the population of the Eu site on the first microbe by 5% (e.g., from 0.462 to 0.512), increase the population of the Yb site on the second microbe by 5% (e.g., from 0.370 to 0.420), and increase the population fraction of the La site on the third microbe by 5% (e.g., from 0.168 to 0.218). (3–6) Reduction in biosorption/desorption cycles needed to achieve a Eu purity of 80%, 90%, 95%, and 99%. This table can be reproduced with the programs Fig-5A.py and Fig-5B.py in the ree-purification repository.^54^

### Feasibility of industrial implementation

A key challenge in industrial implementation of biosorption-based lanthanide separation will be the reuse of biomass for multiple separation steps. At the time of writing, it is uncertain how many times microbial biomass (e.g., from *Shewanella oneidensis* or *V. natriegens*) can be reused. We do know that *Caulobacter crescentus* engineered to surface-display LBTs have been reused three times without loss.^30^ Furthermore, we know that the genome edits to *V. natriegens* that we performed to improve its biosorption selectivity did not significantly impact its fitness.^14^ Finally, it appears that microbial biomass does not need to be alive to perform selective biosorption (Figure S3A). We take these three data points as indicators that biomass can be reused many times, although we must stress that this needs to be tested. Finally, we have demonstrated that we can produce a strain of *V. natriegens* with improved ytterbium tolerance (Figure S3B), suggesting that we can improve biomass survivability through successive rounds of directed evolution if this becomes necessary.

### Model synthesis

In this article, we present three models of lanthanide separation by repeated biosorption and desorption. Model 1 describes a microbe with a single type of binding site (most applicable to microbes that surface-display immobilized chelators). Model 2 describes a microbe with three selective binding sites, each one with a preference for one metal. Model 3 uses three microbes in series, the first to bind the target metal and the second and third to remove contaminants.

Despite the small size of changes to biosorption preference (≈1% to 4%) caused by disruptions to single genomic loci observed in our recent work,^13^ model 1 predicts that these changes might produce large reductions in the length of a repeated enrichment process for individual lanthanides (Figure 3; Table 3). If *S. oneidensis* behaves like it has a single type of binding site (model 1; this does not mean that *S. oneidensis* only has one physical species of binding site, only that its separation factors for multiple lanthanides remains constant as the external concentrations change) (STAR Methods, Figures 1A, 2, and 3), then even the mutants that we have right now^13^^,^^14^ could be used to reduce the length of the separation process for Eu by up to ≈25% (Figure 3E; Table 3). If edits to multiple genomic loci could be combined in a single strain, then it is possible that the length of the purification process could be reduced even more.

Furthermore, we believe that this genetic engineering could be easily achieved. We anticipate that industrialization of lanthanide separation by biosorption (if it works at all) will use the highly engineerable microbe *V. natriegens*. In our recent work on engineering *V. natriegens*, we used directed evolution (rather than targeted edits) to increase biosorption selectivity. We believe that changes in biosorption were due to changes in ≈16 genomic sites.^14^ Multiplex genome editing by natural transformation was recently used to produce ≈9 simultaneous genomic edits in *V. natriegens*.^58^ More recently, multiplex automated genome engineering with conventional transformation technology (i.e., electroporation) was used to produce simultaneous edits to up to 10 genomic sites in the industrially useful microbes *Corynebacterium glutamicum* and *Bacillus subtilis*.^59^

On the other hand, if a microbe has multiple binding sites, each with a relatively strong preference for individual lanthanides (Figures 1, S1, and 4; Table 4), model 2 predicts that purification by repeated biosorption and elution becomes difficult without large genetic engineering efforts (Figures 4C and 4D).

Even though small edits to a single microbe with multiple strongly selective sites will not be useful in purifying an individual lanthanide, we hypothesized that by combining multiple microbes together in a purification process, we could achieve high purity (model 3; Figures 1B, S2, and 5; Table 5). This system enriches for M_1_ in the first column (e.g., Eu), and then removes M_2_ and M_3_ in the second and third columns, respectively. Model 3 predicts that this allows us to reach high purity of Eu by repeated biosorption and elution without heroic genetic engineering efforts. In order to reach 99% purity, we estimate that population fraction of the important site in each microbe must be incremented by ≈15% (site 1 in microbe 1 from 0.462 to 0.612, site 2 in microbe 2 to 0.52, and site 3 in microbe 3 to 0.318).

While the models presented in this article are idealized and contain some arbitrary assumptions, we believe that they give a valuable intellectual framework to build, operate, and troubleshoot biosorption-based REE separation systems. Improving this model will likely go hand in hand with operation of real-world REE separation systems.

Could a lanthanide separation system based around selective biosorption and desorption be implemented industrially? We would be lying if we said we were certain. At the time of writing, there is only a small body of literature on lanthanide separation by selective biosorption. But, we can say with some certainty that any new technology needs to outperform existing technologies to be competitive. Thus, the first thing that we needed to address was if the separation performance of biosorption could be meaningfully improved with achievable genetic engineering. Now that we have this answer, we feel that we can move onto questions of economics and industrial implementation.

Finally, is biosorption the best strategy for individual REE separation? There are at least three possible principles under which a biological lanthanide separation system could operate: selective chelation (e.g., lanmodulin^37^), selective hyper-accumulation (e.g., *Methylorubrum extorquens* Evo^36^), and selective biosorption (this work, Bonificio et al.,^47^ and Medin et al.^14^). Selective chelation and hyper-accumulation both have much higher reported separation factors (especially between light and heavy, and medium and heavy lanthanides) than selective biosorption (with the exception of adjacent heavy lanthanides, where only values for selective biosorption are reported to our knowledge^14^). However, separation factors are not the only consideration involved in building a functioning separation system. Cost and ease of operation are vitally important too. For example, extracting lanthanides from a hyper-accumulating microbe poses a significant challenge to economical recovery. Meanwhile, selective chelation requires that the chelator protein remains intact for many separation operations. On the other hand, selective biosorption allows for easy recovery and does not appear to require live biomass (Figure S3). To us, this indicates that it will be difficult to predict which technology will ultimately prove most useful and be widely adopted. The answer will have to be found by competition in the real world. However, the results presented here, that even small genetic edits can dramatically improve the separation performance of a selective biosorption system, tell us that it is worth trying to implement this system in the real world.

### Limitations of the study

We see at least four significant limitations of this study. First, our models of lanthanide separation make very simplified assumptions about the nature of biosorption. The single-site model assumes that biosorption can be described by only three dissociation constants, while the multi-site model assumes that it can be described by only nine dissociation constants. Furthermore, we assume highly idealized solution conditions containing only three lanthanides, and no competing metals.

Second, this study provides no experimental validation. While theoretical predictions are made, we are not yet able to say how well the predictions of these models will translate to the real world and performance in industrial settings. Furthermore, experimental data in the scientific literature on separation by repeated biosorption are almost non-existent.

Third, we make the assumption that biomass can be extensively reused for biosorption. But, the ability to reuse microbial biomass for multiple separation cycles without degradation is uncertain and needs experimental verification.

Finally, our study does not address the economic viability or industrial scalability of the proposed biosorption-based separation methods. Furthermore, we do not make comparisons with existing industrial lanthanide separation processes.

These limitations highlight the need for further experiments and refinement of the models to assess the practicality and scalability of the proposed biosorption-based lanthanide separation technologies. We hope that this theoretical study provides the impetus to make these measurements.

## Resource availability

### Lead contact

Further information and requests for resources and reagents should be directed to and will be fulfilled by the lead contact, Buz Barstow (bmb35@cornell.edu).

### Materials availability

This study did not generate any unique reagents.

### Data and code availability

• All original code used in this study has been deposited on GitHub at https://github.com/barstowlab/ree-purification and archived on Zenodo at https://doi.org/10.5281/zenodo.8397315.
• All data underlying the figures and analyses in this study have been deposited on GitHub at https://github.com/barstowlab/ree-purification and archived on Zenodo at https://doi.org/10.5281/zenodo.8397315.
• Any additional information required to reanalyze the data reported in this paper is available from the lead contact upon request.

## Acknowledgments

S.M. was supported by a Cornell Presidential Life Sciences Graduate Fellowship. This work was supported by 10.13039/100007231Cornell University startup funds, an Academic Venture Fund award from the 10.13039/100013044Atkinson Center for Sustainability at 10.13039/100007231Cornell University, a Career Award at the Scientific Interface from the 10.13039/100000861Burroughs Wellcome Fund to B.B., 10.13039/100006133ARPA-E award DE-AR0001341 to B.B., 10.13039/100006602Air Force Research Laboratory award FA8750-22-2-0500 to B.B., NSF-TIP award 2228821 to B.B., and a gift from Mary Fernando Conrad and Tony Conrad to B.B.

## Author contributions

Conceptualization, C.A., S.M., and B.B.; methodology, C.A., S.M., and B.B.; investigation, C.A., S.M., J.L.A., B.D., L.E., E.E., C.K., J.J.L., T.J.S., D.S., Z.T., M.X., K.Z., and B.B.; writing – original draft, J.L.A., B.D., E.E., C.K., J.J.L., T.J.S., D.S., Z.T., M.X., K.Z., and B.B.; writing – review and editing, B.B.; funding acquisition, B.B.; resources, M.R., E.G., M.W., and B.B.; supervision, B.B.; data curation, B.B.; visualization, B.B.

## Declaration of interests

S.M. is a co-founder of (B.B. is an unpaid contributor to) REEgen, Inc., which is developing genetically engineered microbes for REE bio-mining.

## Declaration of generative AI and AI-assisted technologies in the writing process

During the preparation of this work, the author(s) used ChatGPT, Cornell Sandbox AI, Elicit, and Perplexity to assist in literature review in response to reviewer comments, to speed generation of computer code, to format references in the supplementary material to meet *iScience* requirements, and to shorten text to meet length requirements of *iScience*. After using these tools, we reviewed and edited the content as needed and take full responsibility for the content of the publication.

## STAR★Methods

### Key resources table

**Table:** 

REAGENT or RESOURCE	SOURCE	IDENTIFIER
**Software and algorithms**		

Python 3.12.7	Python Software Foundation	https://www.python.org
iPython 8.25.0	The iPython Development Team	https://www.ipython.org
Model code	This paper	https://doi.org/10.5281/zenodo.8397315 https://github.com/barstowlab/ree-purification

### Method details

All computer models were performed with iPython version 8.25.0 with Python 3.12.7 (for details, see key resources table). Graphs were produced with DataGraph, and graphics were produced with Adobe Illustrator.

We developed custom computer codes written in Python 3 that calculate the lanthanide separation by microbes with a single type of binding site (Model 1), and lanthanide separation by microbes with three different types of binding site (Models 2 and 3). These models repeatedly solve a set of chemical equilibrium equations for each pass through an immobilized microbe purification column and calculate the concentration of lanthanides in the free and bound phases at each purification step. The solution to the bound phase is used as the input for the subsequent separation step until the desired purity is achieved. Flow charts for Models 1, 2, and 3 are shown in Figures 2, S1, and S2 respectively.

#### Model for separation system using a single type of binding site

In the simplest model of the lanthanide separation scheme, we consider biomass that contains a single type of binding site (results shown in Figure 2; Table 2). The binding site has dissociation constants *K*_D,1_, *K*_D,2_, and *K*_D,3_ for M_1_, M_2_, and M_3_ respectively. After equilibration, the bound and free concentrations of M_1_ (*c*_M1,f_, *c*_M1,b_) and the concentration of free binding sites (*c*_B,f_) are related by dissociation constant *K*_D,1_,

(Equation 12) $$K_{D,1}=\frac{\left(c_{M1,f}{\;c}_{B,f}\right)}{c_{M1,b}}\text{.}$$

Likewise, for metals 2 and 3,

(Equation 13) $$K_{D,2}=\frac{\left(c_{M2,f}{\;c}_{B,f}\right)}{c_{M2,b}}\text{,}$$

(Equation 14) $$K_{D,3}=\frac{\left(c_{M3,f}{\;c}_{B,f}\right)}{c_{M3,b}}\text{.}$$

The number of moles of each metal atom in the bound and free phases is just the concentration multiplied by the system volume. For example,

(Equation 15) $$n_{M1,f}=c_{M1,f}{\;V}_{\text{load}}\text{,}$$

(Equation 16) $$n_{M1,b}=c_{M1,b}\;V_{\text{load}}\text{,}$$

The total number of binding sites is equal to the number of free and bound sites,

(Equation 17) $$n_{B,T}=V_{\text{load}}\left(c_{B1,f}+c_{B1,b}\right)\text{.}$$

The total number of each metal is just the sum of bound and free ions,

(Equation 18) $$n_{M1,T}=V_{\text{load}}\left(c_{M1,f}+c_{M1,b}\right)\text{,}$$

(Equation 19) $$n_{M2,T}=V_{\text{load}}\left(c_{M2,f}+c_{M2,b}\right)\text{,}$$

(Equation 20) $$n_{M3,T}=V_{\text{load}}\left(c_{M3,f}+c_{M3,b}\right)\text{.}$$

Parameters for the solution of this system of 8 equations were managed with a custom code (the function BindState_3Metals_1Site in ConcentrationSolverUtils8^54^) written in Python, and the equations were solved numerically using SymPy.^60^ After the solution to the equations are found, the system is re-started, using just the bound metal ions as the new total metal ions for the next round (*n*_M1,b_, *n*_M2,b_, and *n*_M3,b_).

The ratio of concentrations of each metal in the free and bound phases are called distribution coefficients,

(Equation 21) $$D_{1}=\frac{c_{M1,f}}{c_{M1,b}}\text{,}$$

(Equation 22) $$D_{2}=\frac{c_{M2,f}}{c_{M2,b}}\text{,}$$

(Equation 23) $$D_{3}=\frac{c_{M3,f}}{c_{M3,b}}\text{.}$$

Furthermore, the ratio of distribution coefficients between metals are called separation factors,

(Equation 24) $$\alpha_{1}^{2}=\frac{D_{2}}{D_{1}}\text{,}$$

(Equation 25) $$\alpha_{1}^{3}=\frac{D_{3}}{D_{1}}\text{,}$$

(Equation 26) $$\alpha_{2}^{3}=\frac{D_{3}}{D_{2}}\text{.}$$

For a system containing only a single type of binding site (or at least binding sites that can all be described by a single set of dissociation constants) the separation factors can be expressed simply. For example,

(Equation 27) $$\alpha_{1}^{2}=\frac{D_{2}}{D_{1}}\text{,}=\frac{\left(c_{M2,f}/c_{M2,b}\right)}{\left(c_{M1,f}/c_{M1,b}\right)}\text{,}=\frac{\left(K_{D,2}/c_{B,f}\right)}{\left(K_{D,1}/c_{B,f}\right)}=\frac{K_{D,2}}{K_{D,1}}\text{.}$$

Likewise, when there is only one type of binding site,

(Equation 28) $$\alpha_{1}^{3}=\frac{K_{D,3}}{K_{D,1}}\text{,}$$

(Equation 29) $$\alpha_{3}^{2}=\frac{K_{D,2}}{K_{D,3}}\text{.}$$

As a result of this, the separation behavior of the system depends on the ratio of dissociation constants rather than their absolute magnitude. Furthermore, as the separation factor depends upon intrinsic molecular properties, it remains constant as the concentrations of metals and binding sites change (see Figure 2D for an example of this; Figure 3D for an example of where it does not).

As the separation process proceeds, the number of binding sites in the column is adjusted to be equal to half the total number of ions in the load solution (this could be accomplished by sliding the biosorption material out of the loaded solution). Thus,

(Equation 30) $$n_{B,T}=0.5\times n_{\text{MT}}$$

Finally, to compare with experiment, we calculate the free and bound fractions of each metal in each phase. The proportion of each metal in the free (liquid) fraction,

(Equation 31) $$f_{M1,f}=\frac{n_{M1,f}}{\left(n_{M1,f}+n_{M2,f}+n_{M3,f}\right)}\text{,}$$

(Equation 32) $$f_{M2,f}=\frac{n_{M2,f}}{\left(n_{M1,f}+n_{M2,f}+n_{M3,f}\right)}\text{,}$$

(Equation 33) $$f_{M3,f}=\frac{n_{M3,f}}{\left(n_{M1,f}+n_{M2,f}+n_{M3,f}\right)}\text{.}$$

and in the bound fractions,

(Equation 34) $$f_{M1,b}=\frac{n_{M1,b}}{\left(n_{M1,b}+n_{M2,b}+n_{M3,b}\right)}\text{,}$$

(Equation 35) $$f_{M2,b}=\frac{n_{M2,b}}{\left(n_{M1,b}+n_{M2,b}+n_{M3,b}\right)}\text{,}$$

(Equation 36) $$f_{M3,b}=\frac{n_{M3,b}}{\left(n_{M1,b}+n_{M2,b}+n_{M3,b}\right)}\text{.}$$

We fit data on the fractions of Eu^13^ (*f*_M1,b_ or *f*_Eu, b_); Yb (*f*_M2,b_ or *f*_Yb, b_); and La (*f*_M3,b_ or *f*_La, b_) bound to wild-type *S. oneidensis* to initialize our model.

#### Model for separation system using three types of binding site

A more complex model of lanthanide separation considers multiple types of binding sites. Here we consider a system that contains three types of binding site, each capable of binding all of the three metals in the system (results shown in Figure 3; Table 3). This system is described by 18 simultaneous equations (in contrast to the 8 equations needed to describe a system with one binding site).

The dissociation constant of site 1 for M_1_,

(Equation 37) $$K_{D1,1}=\frac{\left(c_{M1,f}{\;c}_{B1,f}\right)}{c_{M1,b1}}\text{.}$$

where *c*_B1,f_ is the concentration of free type 1 sites, and *c*_M1,b1_ is the concentration of site 1 molecules bound to M_1_. Likewise, the dissociation constant of site 1 for M_2_,

(Equation 38) $$K_{D1,2}=\frac{\left(c_{M2,f}{\;c}_{B1,f}\right)}{c_{M2,b1}}\text{.}$$

where *c*_M2,b1_ is the concentration of site 1 molecules bound to M_2_. Likewise,

(Equation 39) $$K_{D1,3}=\frac{\left(c_{M3,f}{\;c}_{B1,f}\right)}{c_{M3,b1}}\text{.}$$

where *c*_M3,b1_ is the concentration of site 1 molecules bound to M_3_. Furthermore, for sites 2 and 3,

(Equation 40) $$K_{D2,1}=\frac{\left(c_{M1,f}{\;c}_{B2,f}\right)}{c_{M1,b2}}\text{,}$$

(Equation 41) $$K_{D2,2}=\frac{\left(c_{M2,f}{\;c}_{B2,f}\right)}{c_{M2,b2}}\text{,}$$

(Equation 42) $$K_{D2,3}=\frac{\left(c_{M3,f}\;c_{B2,f}\right)}{c_{M3,b2}}\text{,}$$

(Equation 43) $$K_{D3,1}=\frac{\left(c_{M1,f}{\;c}_{B3,f}\right)}{c_{M1,b3}}\text{,}$$

(Equation 44) $$K_{D3,2}=\frac{\left(c_{M2,f}{\;c}_{B3,f}\right)}{c_{M2,b3}}\text{,}$$

(Equation 45) $$K_{D3,3}=\frac{\left(c_{M3,f}{\;c}_{B3,f}\right)}{c_{M3,b3}}\text{.}$$

The total number of metal 1, 2, and 3 ions,

(Equation 46) $$n_{M1,T}=V_{\text{load}}\left(c_{M1,f}+c_{M1,b1}+c_{M1,b2}+c_{M1,b3}\right)\text{,}$$

(Equation 47) $$n_{M2,T}=V_{\text{load}}\left(c_{M2,f}+c_{M2,b1}+c_{M2,b2}+c_{M2,b3}\right)\text{,}$$

(Equation 48) $$n_{M3,T}=V_{\text{load}}\left(c_{M3,f}+c_{M3,b1}+c_{M3,b2}+c_{M3,b3}\right)\text{.}$$

The total number of binding sites of type 1, 2 and 3,

(Equation 49) $$n_{B1,T}=V_{\text{load}}\left(c_{B1,f}+c_{M1,b1}+c_{M2,b1}+c_{M3,b1}\right)\text{,}$$

(Equation 50) $$n_{B2,T}=V_{\text{load}}\left(c_{B2,f}+c_{M1,b2}+c_{M2,b2}+c_{M3,b2}\right)\text{,}$$

(Equation 51) $$n_{B3,T}=V_{\text{load}}\left(c_{B3,f}+c_{M1,b3}+c_{M2,b3}+c_{M3,b3}\right)\text{.}$$

Finally, the concentration of bound sites of type 1, 2, and 3,

(Equation 52) $$c_{B1,b}=c_{M1,b1}+c_{M2,b1}+c_{M3,b1}\text{,}$$

(Equation 53) $$c_{B2,b}=c_{M1,b2}+c_{M2,b2}+c_{M3,b2}\text{,}$$

(Equation 54) $$c_{B3,b}=c_{M1,b3}+c_{M2,b3}+c_{M3,b3}\text{.}$$

The last three Equations 52, 53, and 54 are not strictly necessary but do help to stabilize the numerical solution.

Parameters for the solution of this system of 18 equations were managed with a custom code (the function BindState_3Metals_3Sites in ConcentrationSolverUtils8^54^) written in Python, and the equations were solved numerically using SymPy.^60^

The distribution coefficients can be calculated similarly to Equations 21, 22, and 23, but are complicated by the fact that each metal binds to more than one site. For example,

(Equation 55) $$D_{1}=\frac{c_{M1,f}}{c_{M1,b1}+c_{M1,b2}+c_{M1,b3}}\text{.}$$

Substituting in Equations 37, 38, 39, 40, 41, 42, 43, 44, and 45,

(Equation 56) $$\begin{array}{ll} D_{1} & =\frac{c_{M1,f}}{\frac{c_{M1,f}}{\left(c_{B1,f}{\;K}_{D1,1}\right)}+\frac{c_{M1,f}}{\left(c_{B2,f}{\;K}_{D2,1}\right)}+\frac{c_{M1,f}}{\left(c_{B3,f}{\;K}_{D3,1}\right)}}\text{,}\\ & =\frac{1}{\frac{1}{\left(c_{B1,f}{\;K}_{D1,1}\right)}+\frac{1}{\left(c_{B2,f}{\;K}_{D2,1}\right)}+\frac{1}{\left(c_{B3,f}{\;K}_{D3,1}\right)}}\text{.} \end{array}$$

As in the previous section, we compare our results to experiment by comparing the calculated free and bound fractions of each metal in each phase.

### Quantification and statistical analysis

No statistical tests were used in the preparation of this article.

## Supplemental information

Document S1. Figures S1–S3, Tables S1 and S2, and Note S1
