# Supplementary material for: Constraints on lanthanide separation by selective biosorption
Source: iScience. 2025 Mar 27;28(5):112095. doi: 10.1016/j.isci.2025.112095 (PMC12020885; doi:10.1016/j.isci.2025.112095)
Supplement: Document S1. Figures S1–S3, Tables S1 and S2, and Note S1 [file mmc1.pdf]

iScience, Volume *28*

Supplemental information

Constraints on lanthanide separation by selective biosorption

Carter Anderson, Sean Medin, James L. Adair, Bryce Demopoulos, Liad Elmelech, Emeka Eneli, Chloe Kuelbs, Joseph J. Lee, Timothy J. Sheppard, Deniz Sinar, Zacharia Thurston, Mingyang Xu, Kang Zhang, and Buz Barstow

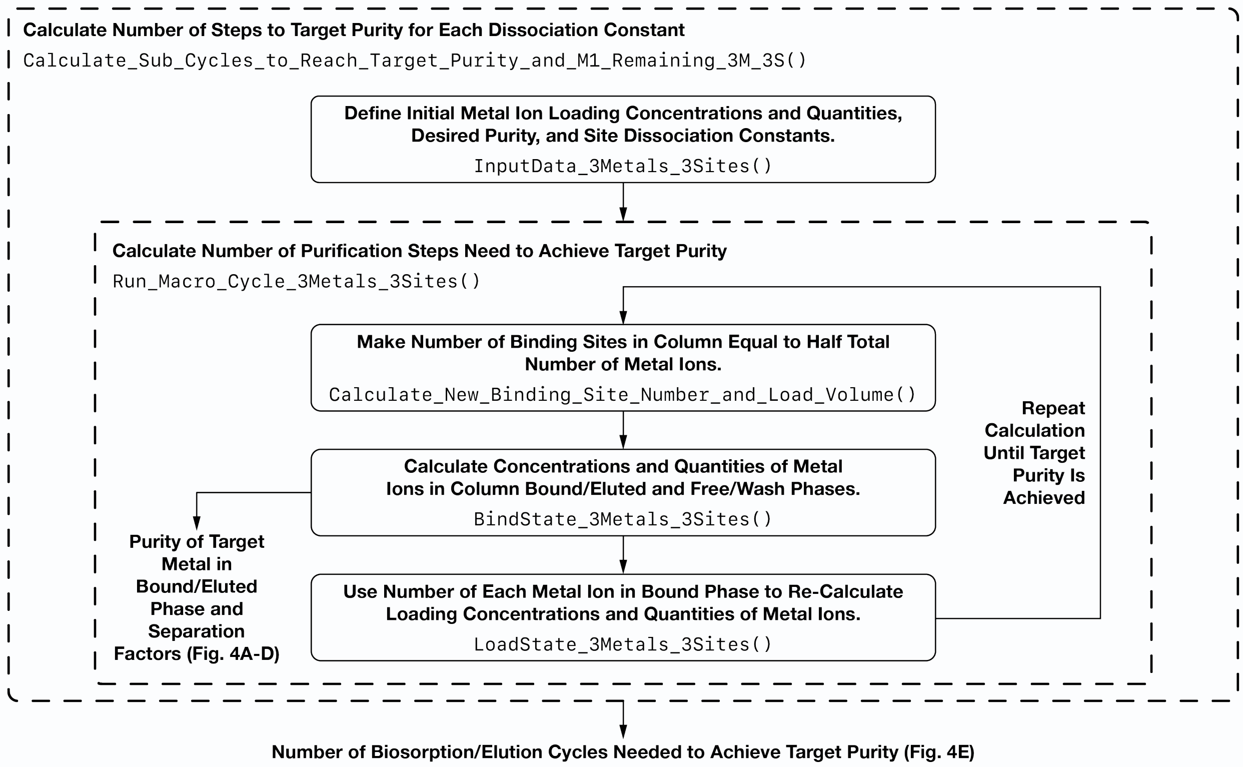

Figure S1. Calculation flow chart for the three-site, single-microbe model (Model 2), related to **Figures 1**, **2**, **4**, **S2**, and **STAR Methods**. This flow chart highlights important calculation steps in the single-site, three-microbe model (**Figures 1A** and **4**) and the functions used to implement them in the CONCENTRATIONSOLVERUTILS9.PY module included in the REE-PURIFICATION repository [S1]. Corresponding flow charts for the single-site, single-microbe model; and the three-site, three-microbe model are shown in **Figures 2** and **S2**.

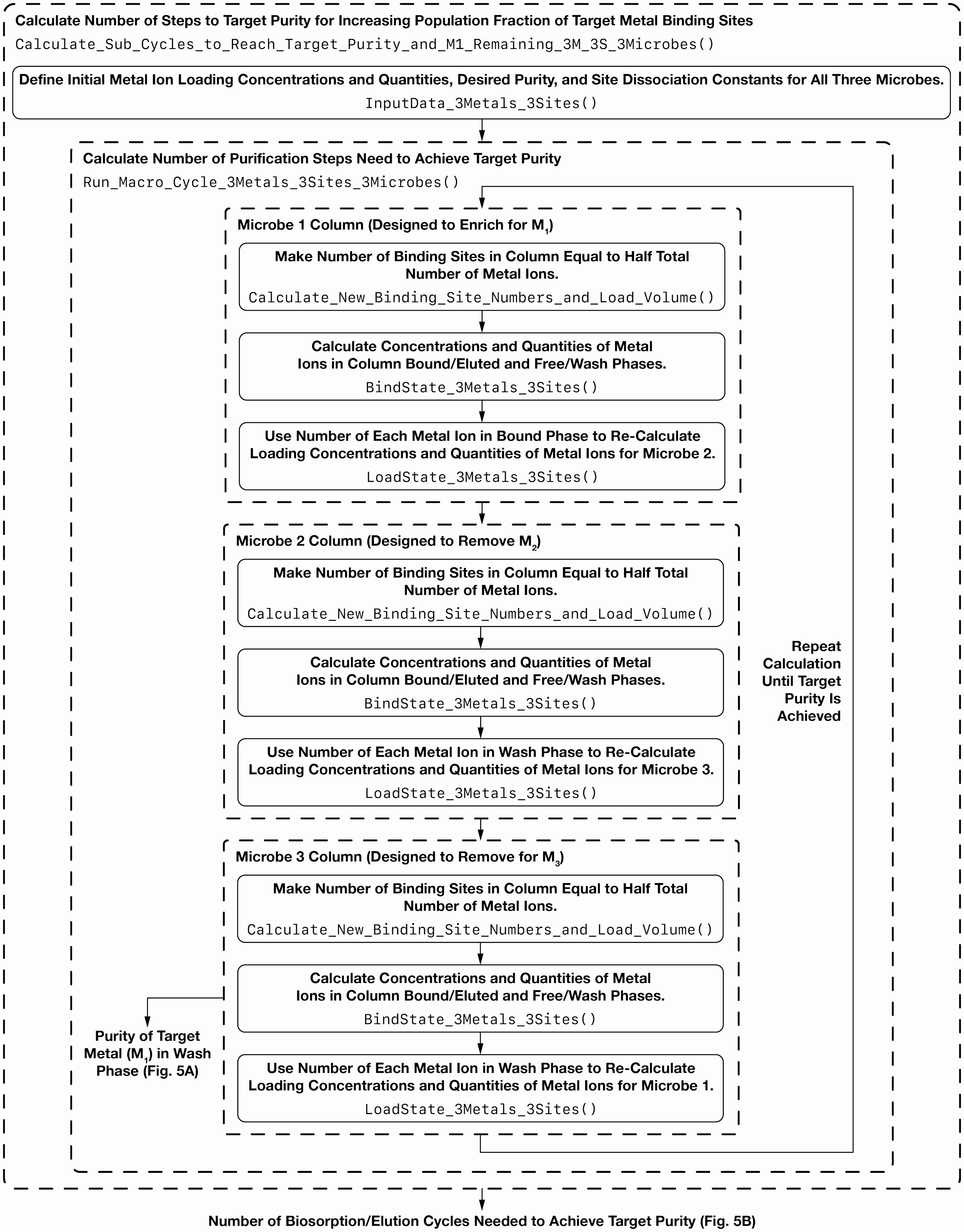
Calculate Number of Steps to Target Purity for Increasing Population Fraction of Target Metal Binding Sites
Calculate_Sub_Cycles_to_Reach_Target_Purity_and_M1_Remaining_3M_3S_3Microbes()
Define Initial Metal Ion Loading Concentrations and Quantities, Desired Purity, and Site Dissociation Constants for All Three Microbes.
InputData_3Metals_3Sites()
Calculate Number of Purification Steps Need to Achieve Target Purity
Run_Macro_Cycle_3Metals_3Sites_3Microbes()
Microbe 1 Column (Designed to Enrich for M_1)
Make Number of Binding Sites in Column Equal to Half Total Number of Metal Ions.
Calculate_New_Binding_Site_Numbers_and_Load_Volume()
Calculate Concentrations and Quantities of Metal Ions in Column Bound/Eluted and Free/Wash Phases.
BindState_3Metals_3Sites()
Use Number of Each Metal Ion in Bound Phase to Re-Calculate Loading Concentrations and Quantities of Metal Ions for Microbe 2.
LoadState_3Metals_3Sites()
Microbe 2 Column (Designed to Remove M_2)
Make Number of Binding Sites in Column Equal to Half Total Number of Metal Ions.
Calculate_New_Binding_Site_Numbers_and_Load_Volume()
Calculate Concentrations and Quantities of Metal Ions in Column Bound/Eluted and Free/Wash Phases.
BindState_3Metals_3Sites()
Use Number of Each Metal Ion in Wash Phase to Re-Calculate Loading Concentrations and Quantities of Metal Ions for Microbe 3.
LoadState_3Metals_3Sites()
Repeat Calculation Until Target Purity Is Achieved
Microbe 3 Column (Designed to Remove for M_3)
Make Number of Binding Sites in Column Equal to Half Total Number of Metal Ions.
Calculate_New_Binding_Site_Numbers_and_Load_Volume()
Calculate Concentrations and Quantities of Metal Ions in Column Bound/Eluted and Free/Wash Phases.
BindState_3Metals_3Sites()
Purity of Target Metal (M_1) in Wash Phase (Fig. 5A)
Use Number of Each Metal Ion in Wash Phase to Re-Calculate Loading Concentrations and Quantities of Metal Ions for Microbe 1.
LoadState_3Metals_3Sites()
Number of Biosorption/Elution Cycles Needed to Achieve Target Purity (Fig. 5B)

Figure S2. Calculation flow chart for the three-site, three-microbe model (Model 3), related to **Figures 1**, **2**, **5**, **S1**, and **STAR Methods**. This flow chart highlights important calculation steps in the three-site, three-microbe model (**Figures 1B** and **5**) and the functions used to implement them in the CONCENTRATIONSOLVERUTILS9.PY module included in the REE-PURIFICATION repository [S1]. Corresponding flow charts for the single-site, single-microbe model; and the three-site, single-microbe model are shown in **Figures 2** and **S1** respectively.

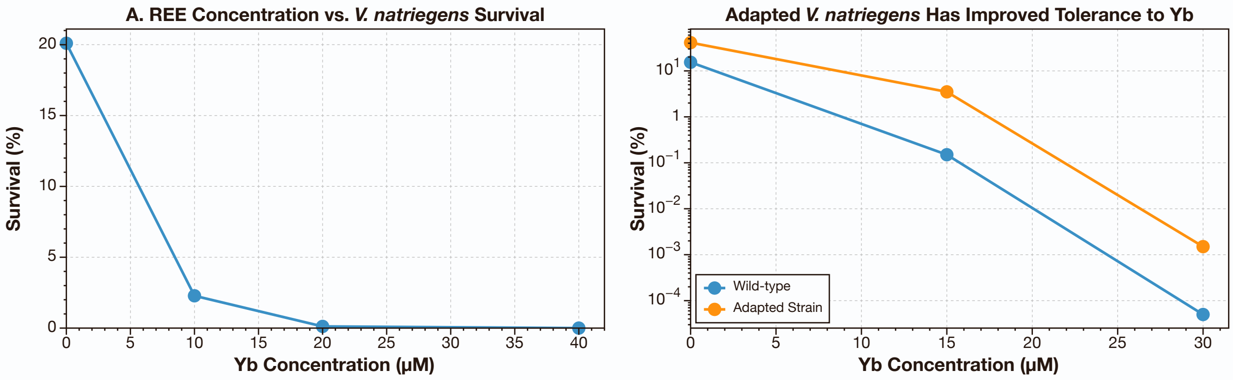

Figure S3. *Vibrio natriegens* has a low survival rate under selective biosorption conditions, related to **Conclusions**. We recently enhanced the selective lanthanide biosorption of the fast-growing microbe *V. natriegens* [S2]. (**A**) Under selective biosorption conditions (20 mM MES buffer, 30 mM total lanthanides), *V. natriegens* has a very low survival rate, suggesting that microbial biomass does not need to be alive to perform selective biosorption. (**B**) However, if biomass survivability becomes an issue, we were able to generate ytterbium-tolerant strains of *V. natriegens*.

Symbol	Unit	Description
α_x^y	#	Separation factor for metals *x* and *y*. $\alpha_x^y = D_y / D_x$.
$c_{\mathrm{B}x,\mathrm{f}}$	Mol L^{-1}	Concentration of binding site x that is unoccupied.
$c_{\mathrm{M}x,\mathrm{b}}$	Mol L^{-1}	Concentration of metal *x* in the bound phase (used in single-site model).
$c_{\mathrm{M}x,\mathrm{b}y}$	Mol L^{-1}	Concentration of metal *x* bound to site *y*.
$c_{\mathrm{M}x,\mathrm{f}}$	Mol L^{-1}	Concentration of metal *x* in the solution phase.
D_x	#	Distribution coefficient for metal *x*. The ratio of Mx in the free and bound phases. $D_x = c_{\mathrm{M}x,\mathrm{f}} / c_{\mathrm{M}x,\mathrm{b}}$.
$f_{\mathrm{B}x}$	#	Fraction of total binding sites in immobilized biomass made up of site *x*.
$f_{\mathrm{M}x,\mathrm{b}}$	#	Fraction of metal *x* (*e.g.*, M_1 or Eu) bound to immobilized biomass.
$f_{\mathrm{M}x,\mathrm{f}}$	#	Fraction of metal *x* in the free (solution) phase.
$K_{\mathrm{D},\,x}$	Mol L^{-1}	Dissociation constant of single binding site for M_x (*e.g.*, $K_{\mathrm{D,\,Eu}}$).
$K_{\mathrm{D}x,\,y}$	Mol L^{-1}	Dissociation constant of binding site *x* for M_y (*e.g.*, $K_{\mathrm{D1,\,Eu}}$).
N_A	molecule mol^{-1}	Avogadro constant.
$n_{\mathrm{B,T}}$	Mol	Number of binding sites on biosorption column.
n_{MT}	Mol	Total number of metal ions.
$n_{\mathrm{M}x,\mathrm{T}}$	Mol	Number of metal *x* (*e.g.*, Eu) ions loaded into separation column.
V_{load}	L	Volume of solution loaded into biosorption column.

Table S1. Symbols list, related to **Table 2** and **STAR Methods**.

Organism	Loading Capacity (mg per g dry weight)	Metal	Reference
Aspergillus niger	31.9 to 97.6	Zn^{2+}	[S3]
Bacillus circulans	5.8 to 26.5	Cd	[S4]
Citrobacter freundii	7.2 to 35.8	Pb	[S5]
Claviceps paspali	31.9 to 97.6	Zn^{2+}	[S3]
Klebsiella pneumoniae	6.2 to 31.9	Pb	[S5]
Penicillium chrysogenum	19.9 to 85.5	Zn^{2+}	[S3]
Saccharomyces cerevisiae	42 to 60	Ag^{+}	[S6]
Saccharomyces cerevisiae	59	Ag	[S7]
Saccharomyces cerevisiae	189	Pb	[S7]
Spirogyra species	133.3	Cu^{2+}	[S8]
Talaromyces emersonii	280	U^{6+}	[S9]

Table S2. Representative values of metal biosorption per unit mass of dry biomass, related to **STAR Methods**.

Supplementary Information Notes

Note S1. Comparison of Experimental Data with Literature Observations of Biosorption, Related to Results and Discussion

We fit data shown in Medin *et al*. [S10] on biosorption by wild-type *S. oneidensis* to the single- and triple-site models of binding (**STAR Methods**) to establish baseline parameters for our predictions of separation behavior by repeated binding and de-binding.

To sanity check the connection between experimental data and our models, we first calculate the lanthanide binding per gram of dry weight of *S. oneidensis* and compare with literature values. For the purposes of this analysis, we will restrict discussion to the low ionic strength, high REE conditions (LH) because these have the highest binding of REE, so we have to worry least about interference from NaCl.

Under the low ionic strength, high REE conditions (LH), the true wild-type *S. oneidensis* binds a total of 35.7 nanomoles (nmol) of lanthanides, from a total of 72 nmol in solution. The volume in these experiments is 400 µL, with an optical density of 0.85. Assuming that there are approximately 10^9 cells per OD per mL of culture, then in each REE-binding experiment there are a total of 3.4×10^8 cells. This corresponds to 6.3×10^7 atoms of lanthanide per cell.

How does the mass of lanthanides bound per unit of dry weight compare with literature values? Under LH conditions *S. oneidensis* binds 8.9 nmol of La (25.4% of the total); 12.4 nmol Yb (35.3%); and 13.8 nmol of Eu (39.3%). These correspond to 3.6 fg La per cell; 6.3 fg Yb per cell; and 6.1 fg Eu per cell. This corresponds to a total of 16.1 fg total lanthanides per cell.

What is the dry weight per cell of *S. oneidensis*? We assume that *S. oneidensis* has a similar dry weight to *E. coli* which ranges 280 fg under 40 minute doubling conditions (BioNumbers ID (BNID) [S11] 103904); 480 fg (with a range of 358 to 622; BNID 102230); to 640 fg (BNID 100009). The average of these values is 470 fg. Thus the approximate loading capacity of *S. oneidensis* is s 16.1 fg $cell^{-1}$ / 470 fg dry weight $cell^{-1} \approx 30$ mg g^{-1} of dry weight. This number is compares very well to literature values of metal binding per unit mass of dry biomass assembled in **Table S2**.

Supplementary References

[S1] Currently available at https://github.com/barstowlab/ree-purification.

[S2] S. Medin *et al.*, "Multiple Rounds of In Vivo Random Mutagenesis and Selection in Vibrio natriegens Result in Substantial Increases in REE Binding Capacity". *ACS Synthetic Biology* 12(12): 3680-3694 (2023). doi:10.1021/acssynbio.3c00484.

[S3] E. Luef, T. Prey, and C. P. Kubicek. Biosorption of zinc by fungal mycelial wastes. *Applied Microbiology and Biotechnology* 34, 688–692 (1991). doi:10.1007/bf00167924.

[S4] E. I. Yilmaz and N. Y. Ensari. "Cadmium biosorption by *Bacillus circulans* strain EB1". *World Journal of Microbiology & Biotechnology* 21, 777–779 (2005). doi:10.1007/s11274-004-7258-y.

[S5] S. Al-Garni. "Biosorption of lead by Gram-ve capsulated and non-capsulated bacteria". *Water SA* 31, 345–350 (2005). doi:10.4314/wsa.v31i3.5224.

[S6] P. Simmons and I.A. Singleton. "A method to increase silver biosorption by an industrial strain of *Saccharomyces cerevisiae*". *Applied Microbiology and Biotechnology* 45, 278–285 (1996). doi:10.1007/s002530050684.

[S7] M. Bustard, and A.P. McHale. "Biosorption of heavy metals by distillery-derived biomass". *Bioprocess Engineering* 19, 351–353 (1998). doi:10.1007/s004490050531.

[S8] V. K. Gupta, A. Rastogi, V. K. Saini, and N. Jain. "Biosorption of copper(II) from aqueous solutions by *Spirogyra species*". *Journal of Colloid and Interface Science* 296, 59–63 (2006). doi:10.1016/j.jcis.2005.08.033.

[S9] L. Bengtsson, B. Johansson, T.J. Hackett, L. McHale, and A.P. McHale. "Studies on the biosorption of uranium by *Talaromyces emersonii* CBS 814.70 biomass". *Applied Microbiology and Biotechnology* 42, 807–811 (1995). doi:10.1007/bf00171965.

[S10] S. Medin *et al*. "Genomic characterization of rare earth binding by *Shewanella oneidensis*". *Scientific Reports* **13**, 15975 (2023). doi:10.1038/s41598-023-42742-6.

[S11] R. Milo *et al*., "BioNumbers - the database of key numbers in molecular and cell biology". *Nucleic Acids Research* **38**: D750-D753 (2010). doi:10.1093/nar/gkp889.
